# Dorsal Cortical Alignment Predicts Functional Outcomes in Proximal Phalangeal Fractures Treated with Intramedullary Headless Compression Screws but Not in Metacarpal Fractures

**DOI:** 10.3390/jcm14134691

**Published:** 2025-07-02

**Authors:** Bert Vanmierlo, Hans Lowyck, Charles Matthys, Tim Vanmierlo, Joris Duerinckx, Bert O. Eijnde

**Affiliations:** 1 Department of Orthopaedics and Traumatology, AZ Delta, Deltalaan 1, 8800 Roeselare, Belgium; hans.lowyck@azdelta.be; 2 Department of Cardio and Organ Systems, Hasselt University, Martelarenlaan 42, 3500 Hasselt, Belgium; 3 Faculty of Medicine, KU Leuven, Campus Gasthuisberg ON2, Herestraat 49–Bus 400, 3000 Leuven, Belgium; charles.matthys@student.kuleuven.be; 4 Department of Neuroscience, Biomedical Research Institute (Biomed) Hasselt University, Martelarenlaan 42, 3500 Hasselt, Belgium; tim.vanmierlo@uhasselt.be; 5 Division of Translational Neuroscience, Department of Psychiatry and Neuropsychology, Mental Health and Neuroscience (MHeNs) Research Institute, Maastricht University, Minderbroedersberg 4-6, 6211 LK Maastricht, The Netherlands; 6 Department of Orthopaedics and Traumatology, Ziekenhuis Oost-Limburg, Synaps Park 1, 3600 Genk, Belgium; joris.duerinckx@gmail.com; 7 Faculty of Rehabilitation Sciences, Hasselt University, Martelarenlaan 42, 3500 Hasselt, Belgium; 8 SMRC-Sports Medical Research Center, BIOMED-Biomedical Research Institute, Faculty of Medicine & Life Sciences, Hasselt University, Martelarenlaan 42, 3500 Hasselt, Belgium; bert.opteijnde@uhasselt.be; 9 Division of Sport Science, Faculty of Medicine & Health Sciences, Stellenbosch University, Cnr. Ryneveld and Victoria Street, Stellenbosch 7600, South Africa

**Keywords:** intramedullary, phalanx, fracture, metacarpal, screw, osteosynthesis, reduction, fixations, surgery

## Abstract

**Background/Objectives**: Intramedullary headless compression screw (IMHCS) fixation has emerged as a minimally invasive and biomechanically robust method for treating metacarpal and proximal phalangeal fractures. While the clinical outcomes are generally favorable, the impact of anatomical fracture reduction on postoperative function has not been systematically examined. **Methods**: We retrospectively analyzed 69 patients (41 metacarpal, 28 proximal phalanx) treated with IMHCSs between June 2020 and March 2025. Fractures were classified radiographically as reduced or non-reduced. Functional outcomes were assessed using the Total Active Motion (TAM) scoring system. The association between the reduction quality and TAM outcome was analyzed separately for metacarpal and proximal phalangeal fractures using the Fisher–Freeman–Halton exact test. **Results**: All fractures achieved radiographic union. In the metacarpal fractures, 90% of the patients attained good-to-excellent TAM scores, with no statistically significant association between the reduction quality and functional outcome (*p* = 0.1303). In contrast, for the proximal phalangeal fractures, anatomical reduction was significantly associated with superior TAM outcomes (*p* = 0.0014; Cohen’s w = 0.802). The postoperative radiographs in this group revealed smooth dorsal cortical alignment in the patients with good outcomes, suggesting preserved tendon gliding surfaces. **Conclusions**: Anatomical fracture reduction significantly predicts postoperative function in proximal phalangeal fractures treated with IMHCSs. In contrast, metacarpal fractures appear more tolerant of minor malalignment. These findings underscore the importance of achieving cortical continuity in phalangeal fractures to optimize digital biomechanics. A minimal open approach should be considered to ensure proper alignment during IMHCS fixation.

## 1. Introduction

Metacarpal and proximal phalangeal fractures are among the most common injuries encountered in hand surgery, accounting for a significant proportion of upper extremity trauma [1,2]. While many proximal phalangeal fractures can be managed conservatively, surgical intervention is often required for fractures that are displaced, unstable, or rotationally malaligned to restore normal anatomy and function. Unlike minor flexion deficits, rotational malalignment significantly compromises hand function. It is considered unacceptable, as it will not self-correct—whether left untreated or present once rigid fixation is in place—underscoring the critical importance of achieving accurate reduction [3,4,5]. Similar considerations apply to metacarpal fractures. Various fixation techniques have been employed for these fractures, including Kirschner wires (K-wires), interfragmentary screws, and plate constructs [6].

Since the introduction of intramedullary headless compression screws (IMHCSs) for the treatment of delayed unions in phalangeal fractures, this method has emerged as a promising alternative [7]. IMHCS fixation offers a stable construct that allows for early mobilization and restoration of range of motion. The importance of early mobilization was first highlighted in a classic work by Tubiana, who showed that initiating motion as soon as fracture and soft-tissue stability permit helps prevent stiffness in metacarpal and phalangeal injuries [8]. Comparative biomechanical studies have demonstrated that IMHCS fixation offers rigidity, load-to-failure strength, and rotational stability comparable to conventional plate-and-screw constructs [9,10,11].

The use of IMHCSs in metacarpal fractures was first described in 2010 [12]. As with proximal phalangeal fractures, this technique has demonstrated enhanced biomechanical strength compared to traditional plating methods [13,14,15]. Multiple studies have examined its clinical utility, with recent evidence demonstrating comparable or superior outcomes to conventional K-wire techniques [16]. In addition, improved functional outcomes have been reported compared to plate-and-screw osteosynthesis [17]. For proximal phalangeal fractures, a systematic review by Reid et al. reported faster mobilization, earlier returns to work, and fewer complications—particularly stiffness—compared to plates, screws, and percutaneous K-wires [18].

Systematic reviews and large-case series evaluating IMHCS fixation for metacarpal and phalangeal fractures have consistently reported excellent clinical outcomes. Complications have generally ranged between 2.8% and 4.6%, despite some overlap in the included studies [19,20,21].

Despite these favorable outcomes, no studies to date have systematically examined the correlation between the quality of fracture reduction and clinical outcomes in patients treated with IMHCS, either for metacarpal or proximal phalangeal fractures. Independently of the chosen treatment technique, the literature indicates that metacarpal fractures can tolerate minor deformities without functional impairment, provided that rotational alignment is maintained [22,23]. However, evidence is mixed for proximal phalangeal fractures: some studies have linked anatomical reduction to improved outcomes, while others have reported that minor displacement or malalignment does not significantly affect function [24,25,26,27]. As the primary goal in fracture management is to achieve a good and lasting clinical outcome, understanding whether anatomical reduction influences functional recovery is of critical clinical relevance.

This retrospective observational study aims to assess the relationship between fracture reduction quality—defined by standardized radiographic criteria—and postoperative functional outcomes, measured using the Total Active Motion (TAM) scoring system, in patients who have undergone IMHCS fixation for extra-articular metacarpal and proximal phalangeal fractures.

## 2. Materials and Methods

### 2.1. Patient Inclusion

We conducted a single-center retrospective analysis of all patients who underwent osteosynthesis with IMHCSs for metacarpal or proximal phalangeal fractures between June 2020 and March 2025. The inclusion criteria were extra-articular fractures of the metacarpals or proximal phalanges, treated with IMHCS fixation. The exclusion criteria included a history of prior fracture or trauma in the operative region; significant concomitant soft-tissue injury, such as tendon, nerve, or vascular damage; and crush injuries. Fractures with any intra-articular extension were also excluded. Furthermore, patients without follow-up, those with incomplete clinical data, or those lacking serial radiographic imaging to confirm fracture consolidation were excluded from this analysis.

### 2.2. Methodology

In May 2025, a collaborating medical intern, C.M., extracted data from medical records. The collected variables included sex, the affected bone (metacarpal or proximal phalanx), fracture distribution across the fingers/rays, age at the time of trauma, number of screws used per fracture, insertion technique (antegrade or retrograde), range of motion of the affected ray, and any complications. An independent orthopedic surgeon, H.L., who was not involved in the surgical procedures, evaluated the fracture reduction status and time to radiographic consolidation.

In proximal phalanx fractures, a single headless compression screw is typically positioned centrally within the medullary canal and may be inserted either antegrade or retrograde. When two screws are used, two configurations are described. The double-opposing technique involves inserting one screw antegrade and one retrograde, introduced from opposite ends of the phalanx to balance forces and enhance stability. The double-barrel technique, by contrast, uses two screws inserted in the antegrade direction from the base of the proximal phalanx. This method is particularly suited for base fractures, as the screw heads are anchored in the dense subchondral bone of the proximal articular surface, offering excellent purchase and enhanced rotational stability [28]. Early clinical outcomes have been promising, with a case series of 10 patients treated using the double-barrel technique reporting excellent results without complications [29].

The first postoperative radiographs were reviewed to assess fracture reduction. An acceptable alignment was defined as follows: (1) in anteroposterior (AP) view, an angulation less than or equal to 10°; (2) in true lateral view, an angulation less than or equal to 10° at the shaft, an angulation less than or equal to 20° at the juxta-articular region, or an angulation of less than or equal to 45° at the neck of the 5th metacarpal; (3) an area of contact of more than or equal to 50 percent; and (4) no obvious visible rotation [30]. Shortening of the metacarpal was not classified as a non-reduction, as it does not significantly impact digital range of motion. However, it may decrease finger flexion force or grip strength [21,31]. Digital flexion was assessed using the TAM method, adopted by the American Society for Surgery of the Hand (ASSH) in 1983 and later applied in clinical outcome studies such as that by Eberlin et al. [24,32]. An ordinal scoring system was used to classify flexion quality. A score of 0 was assigned when the cumulative flexion was less than 200°, corresponding to poor flexion on the TAM scale. These patients were consistently unable to achieve pulp-to-palm contact during maximal finger flexion. A score of 1 reflected fair flexion, defined as a total arc between 200° and 250°, typically permitting fingertip-to-palm contact without full and symmetrical closure of the affected finger. A score of 2 was assigned when digital flexion reached 250° or more, indicating good to excellent flexion, with the fingertip typically contacting the distal palmar crease. Finally, the correlation between fracture reduction and functional outcome was analyzed separately for metacarpal and proximal phalangeal fractures.

### 2.3. Statistics

All statistical analyses were performed using GraphPad Prism, version 10.0.2 (GraphPad Software, San Diego, CA, USA). Categorical variables—including fracture reduction status and functional outcome scores—were compared using the Fisher–Freeman–Halton exact test implemented in GraphPad Prism due to the relatively small sample sizes and expected cell counts below five in the contingency tables. This test was conducted separately for the metacarpal and proximal phalangeal groups to assess whether anatomical fracture reduction was associated with improved functional outcomes, as measured by the ordinal TAM-based flexion score.

Statistical significance was defined as *p* < 0.05. We report the estimated effect size by reporting Cohen’s w to aid interpretation. No correction for multiple comparisons was applied, as the comparisons were pre-specified and hypothesis-driven.

This study was approved by the institutional ethics committee (approval number B1172025000016).

## 3. Results

A total of 93 fractures treated with intramedullary headless compression screws were initially identified. After applying the inclusion and exclusion criteria, 69 patients were included in the final analysis, comprising 41 with metacarpal fractures and 28 with proximal phalangeal fractures.

A summary of the metacarpal and proximal phalangeal fracture management is presented in Table 1.

In the metacarpal group, the mean age was 35 years (range: 16–74), with 32 males and nine females. The mean time to radiographic consolidation was 46 days (range: 23–140). IMHCS fixation was performed on the second metacarpal in five cases, the third in seven, the fourth in fifteen, and the fifth in fourteen. A retrograde screw insertion was used in 26 cases and an antegrade approach in 15. In all cases, a single screw was used per metacarpal.

In the proximal phalangeal group, the mean age was 39 years (range: 12–73), with 18 males and 10 females. The mean time to consolidation was 41 days (range: 24–89). Fixation was performed using a single screw in twenty phalanges, the double-barrel technique in seven, and double opposing screws in one. Retrograde screw insertion was used in four cases, while an antegrade approach was used in twenty-three. Most phalanges were treated in the fifth ray (*n* = 16), followed by the second and fourth rays (*n* = 5 each) and the third ray (*n* = 2). Radiographic union was achieved in all cases.

**Figure 1:**
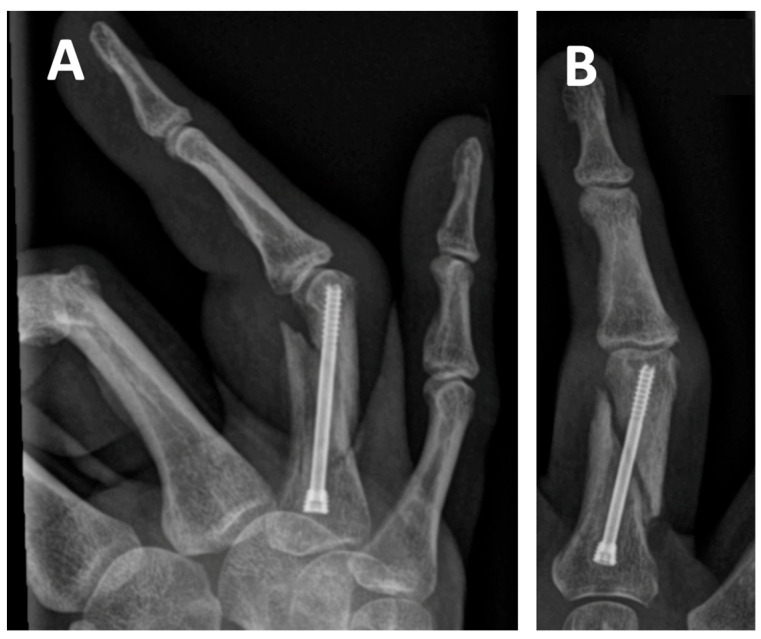
Radiographs of a 61-year-old female patient with a spiral fracture of the proximal phalanx of the right hand. The fracture was treated with antegrade intramedullary headless compression screw (IMHCS) fixation. Panels (**A**,**B**) show postoperative lateral and anteroposterior radiographs of the proximal phalangeal fracture. Due to inadequate fracture reduction, the patient developed mild clinodactyly as a residual deformity.

**Figure 2:**
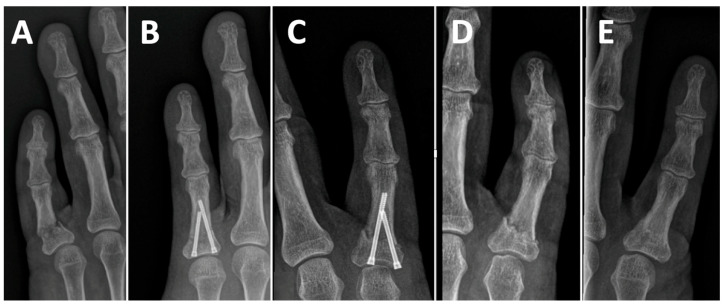
Radiographic sequence of a 60-year-old male patient with a base fracture of the proximal phalanx of the right little finger. (**A**). Anteroposterior radiograph at the time of trauma. (**B**). Postoperative radiograph following double-barrel antegrade intramedullary screw fixation, demonstrating adequate fracture reduction. (**C**). Follow-up radiograph showing fracture collapse and protrusion of the head of the shorter screw. (**D**). Radiograph after removal of the screws. (**E**). Final radiograph demonstrating fracture consolidation.

Two patients in the phalangeal group experienced minor complications. One patient developed a mild (10°) but clinically insignificant clinodactyly (Figure 1). Another patient developed metacarpophalangeal joint arthritis due to screw base protrusion, necessitating screw removal (Figure 2). Cases of limited Total Active Motion were not classified as complications.

Based on TAM scoring, 61% of the proximal phalangeal fractures achieved good to excellent flexion (≥250°) and 18% were classified as fair (200–249°) and 21% as poor (<200°). In contrast, 90% of the metacarpal fractures showed good to excellent outcomes, 7% were fair, and only 2% were poor (Table 2).

Postoperative radiographs were used to categorize the fractures as either reduced or non-reduced. The metacarpal group had no significant correlation between reduction status and postoperative flexion. Both the reduced and non-reduced fractures typically demonstrated excellent ranges of motion. A Fisher–Freeman–Halton exact test confirmed the absence of a statistically significant relationship (*p* = 0.1303; two-tailed, exact test, effect size Cohen’s w = 0.278). (Table 3).

In contrast, reduction status was strongly associated with the outcomes in the phalangeal group. Among the anatomically reduced fractures, 12 patients achieved full digital flexion (score 2), one had moderate restriction (score 1), and none had poor motion (score 0). Only two achieved full flexion among the non-reduced fractures, while three were moderate and eleven poor. This association was statistically significant (*p* = 0.0014; two-tailed, exact test, effect size Cohen’s w = 0.802), supporting the importance of anatomical alignment for optimal digital biomechanics in proximal phalangeal fractures (Table 4).

## 4. Discussion

### 4.1. Metacarpal Fractures

IMHCS fixation has emerged as a valuable alternative to traditional plate-and-screw osteosynthesis and K-wire fixation in metacarpal fractures. A literature review by Beck et al. analyzed multiple studies. It concluded that IMHCS fixation of metacarpal neck and shaft fractures is a safe and effective surgical option associated with superior clinical outcomes, fewer complications, and a reduced need for re-interventions compared to plate fixation or intramedullary nailing [33]. Our findings are consistent with these observations: all patients in our series achieved radiographic consolidation, and only minimal complications were reported. These results support and extend the conclusions of Beck et al. and are in line with systematic reviews by Hug et al. and Morway et al. [19,20].

Furthermore, the majority of patients demonstrated excellent clinical outcomes. However, the fracture reduction was considered non-anatomical in 13 of the 41 metacarpal fractures. Notably, this did not correlate with impaired functional recovery. Statistical analysis showed no significant association between reduction status and postoperative flexion. These findings are in line with the existing literature suggesting that metacarpal fractures can tolerate minor deformities—such as axial shortening of less than 5 mm or angulation of up to 30° in selected rays—without appreciable loss of finger mobility, as long as rotational alignment is preserved [22,23]. Rotational alignment remains the one non-negotiable parameter in metacarpal fractures; even 5–10° of rotation can cause digit overlap and clinically significant impairment [34]. This tolerance is likely due to the role of the metacarpal as a load-bearing lever rather than a direct joint interface and to the greater anatomical distance between the metacarpal shaft and adjacent tendons compared to the phalanges. This spacing reduces the risk of callus-induced adhesions and tenodesis effects during the healing process.

**Figure 3:**
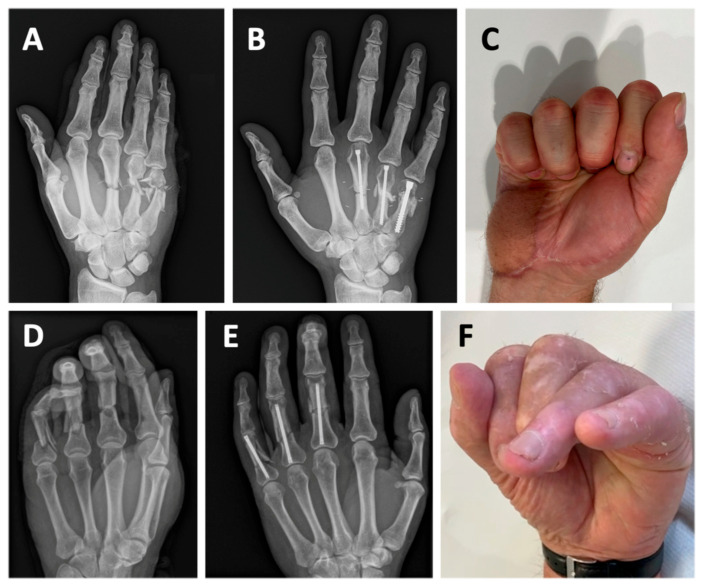
Radiographs and clinical photographs of two patients with complex metacarpal (**A**–**C**) and phalangeal (**D**–**F**) injuries treated using IMHCS fixation. (**A**–**C**): A 42-year-old male with severely comminuted extra-articular fractures of the third, fourth, and fifth metacarpals. (**A**). Preoperative radiograph showing comminuted fractures of the metacarpal shafts. (**B**). Postoperative radiograph at 3 months demonstrating fracture consolidation following retrograde IMHCS fixation. (**C**). Clinical photograph showing full active flexion of the affected fingers. (**D**–**F**): A 73-year-old patient with subtotal amputations of the middle, ring, and little fingers through the proximal phalanges. (**D**). Preoperative radiograph demonstrating comminuted fractures. (**E**). Postoperative radiograph at 3 months showing stable fixation and alignment. (**F**). Clinical photograph illustrating active flexion of the operated fingers. While pulp-to-palm contact was not achieved, the result was functionally satisfactory to the patient.

Consequently, we propose that improving fracture reduction through additional skin incisions or extensive soft-tissue dissection is unnecessary when treating metacarpal fractures with IMHCS fixation. Clinical outcomes appear to remain satisfactory even when fracture reduction is suboptimal. Furthermore, additional dissection may be detrimental, as it can promote scar formation, soft-tissue fibrosis, and tenodesis, potentially compromising finger motion. This is further illustrated by the cases presented in Figure 3, which were excluded from the main study due to significant associated soft-tissue trauma and thus did not meet the inclusion criteria. Both cases illustrate the presence of severe soft-tissue injury combined with complex comminuted fractures. Despite the severity, both achieved bony consolidation, underwent early active mobilization, and ultimately resulted in excellent clinical outcomes. Panels A–C depict a 42-year-old male with severely comminuted extra-articular fractures of the third, fourth, and fifth metacarpals accompanied by complete transection of the corresponding extensor tendons and degloving of the hypothenar region. The fractures were treated using IMHCS fixation, resulting in complete radiographic consolidation within three months and an excellent clinical outcome with full active digital flexion. Despite the extensive soft-tissue injury, this case demonstrates the absence of extensor tendon tenodesis. It highlights the biomechanical stability and clinical utility of IMHCS fixation in complex fracture settings. Panels D–F show a 73-year-old male patient with subtotal amputations of the middle, ring, and little fingers at the level of the proximal phalanges. Fracture fixation was again performed using IMHCSs, leading to a satisfactory functional outcome despite the severity of the initial trauma.

### 4.2. Proximal Phalangeal Fractures

Minimally invasive IMHCS fixation has demonstrated excellent biomechanical properties in treating unstable phalangeal fractures. In a biomechanical study, Miles et al. compared IMHCSs to plate-and-screw fixation and found comparable performance in terms of load to failure and rigidity [10]. Similarly, Rausch et al. compared K-wires, plates, and compression screws, finding that IMHCSs provided the most stable constructs under bending forces. The plate-and-screw fixation performed best under distraction testing, while no significant differences were observed in rotational stability between the IMHCSs and the plating. The K-wire fixation performed significantly worse across all three testing modalities [11].

Plate-and-screw osteosynthesis for phalangeal fractures has been associated with high complication and reoperation rates. A retrospective study of 181 fractures reported reoperation rates of 42% for plate fixation compared with 25% for K-wires and 15% for screws, mainly due to stiffness and the need for hardware removal [35]. Another study showed a 57% major complication rate in 64 phalangeal fractures treated with plates [36], while even low-profile plating systems had reoperation rates of nearly 40% [37]. These data further support the use of low-profile, intramedullary devices like IMHCSs.

The literature on proximal phalangeal fracture management presents diverging views regarding the importance of anatomical reduction in determining clinical outcomes. Several studies suggest that minor displacement or malalignment does not significantly impact function. Alagar et al. reported that in a cohort of patients treated with closed reduction and percutaneous pinning, only 35% achieved anatomical reduction. Yet most attained good to excellent early functional outcomes [25]. Similarly, Singh et al. evaluated 84 cases of closed proximal phalangeal fractures managed conservatively or surgically. Although malunions occurred in both groups, good to excellent results were seen in 89% and 92% of the cases, implying that slight deformities may be functionally tolerated [26]. Eberlin et al. demonstrated favorable outcomes using periarticular pinning techniques even without perfect reduction [24].

Conversely, other authors have emphasized the critical role of anatomical reduction. In a biomechanical context, Ibáñez et al. demonstrated that IMHCS fixation provides superior stability compared to lateral plating, especially when accurate reduction is achieved, highlighting the mechanical importance of anatomical alignment [9]. Heifner and Rubio advocated meticulous reduction and stable fixation to optimize functional recovery and facilitate early mobilization [27]. This viewpoint is particularly relevant to our findings. In our cohort of proximal phalangeal fractures treated with IMHCSs, we observed a 100% union rate, with one patient developing mild clinodactyly and another requiring hardware removal. Moreover, a significant correlation was noted between the quality of fracture reduction and functional outcomes. Patients with non-anatomically reduced fractures were more likely to have impaired ranges of motion. This is likely due to the proximity of the flexor tendons—and especially the extensor apparatus—to the phalangeal shaft, which predisposes them to adhesion formation resulting from callus development, a phenomenon more common in cases of non-anatomical fracture reduction.

**Figure 4:**
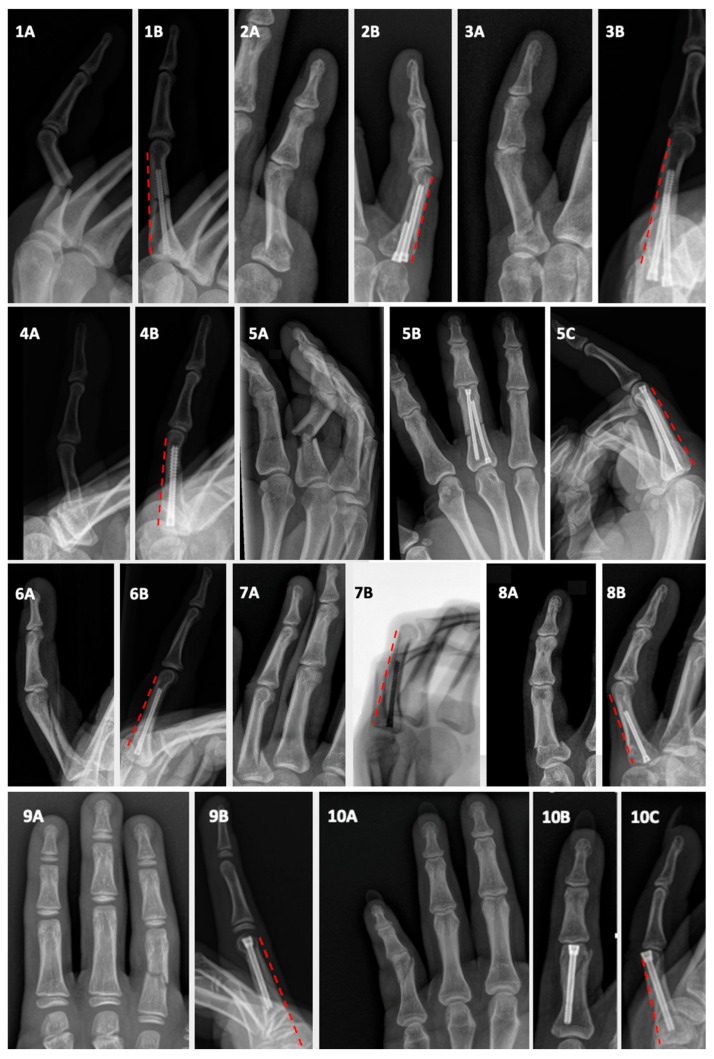
Preoperative and postoperative images of eight patients with anatomically reduced proximal phalangeal fractures with good to excellent TAM. For each patient, panel (**A**) shows the preoperative radiograph and panels (**B**,**C**) show the postoperative true lateral view. In all cases, the dorsal cortex is perfectly aligned without any step-offs (red dot lines), resulting in a smooth gliding surface for the extensor apparatus. (**1A**,**B**). A 31-year-old male with a transverse diaphyseal fracture of the right index finger. (**1A**). Preoperative true lateral view. (1B). Postoperative true lateral view showing antegrade insertion of a single partially threaded IMHCS. (**2A**,**B**). 56-year-old female with a base fracture of the left little finger. (**2A**). Preoperative posteroanterior (PA) view. (**2B**). Postoperative true lateral view showing two partially threaded IMHCSs inserted antegrade using the double-barrel technique. (**3A**,**B**). A 44-year-old female with a base fracture of the left little finger. (**3A**). Preoperative oblique view. (**3B**). Postoperative true lateral view showing double-barrel antegrade screw fixation. (**4A,B**). A 60-year-old female with a proximal-third fracture of the left little finger. (**4A**). Preoperative true lateral view. (**4B**). Postoperative true lateral view showing a single fully threaded IMHCS inserted antegrade. (**5A**–**C**). A 48-year-old male with a diaphyseal fracture of the right middle finger. (**5A**). Preoperative oblique view. (**5B**). Postoperative PA view. (**5C**). Postoperative true lateral view showing two partially threaded IMHCSs inserted using the technique of the double-opposing screws. (**6A**,**B**). A 15-year-old male with a spiral fracture of the left ring finger. (**6A**). Preoperative PA view. (6B). Postoperative true lateral view showing antegrade insertion of a single fully threaded IMHCS. (**7A,B**). A 20-year-old male with a base fracture of the left ring finger. (**7A**). Preoperative and (**7B**). postoperative true lateral views showing antegrade insertion of a single fully threaded IMHCS. (**8A**,**B**). A 41-year-old female with a base fracture of the left little finger. (**8A**). Preoperative PA view. (**8B**). Postoperative true lateral view showing antegrade insertion of a single partially threaded IMHCS. (**9A**,**B**). A 12-year-old male with a straight diaphyseal fracture of the right ring finger. (**9A**). Preoperative view. (**9B**). Postoperative true lateral view showing retrograde insertion of a single fully threaded IMHCS. (**10A**–**C**). A 15-year-old male with a distal metaphyseal fracture of the right little finger. (**10A**). Preoperative PA view. (**10B**). Postoperative PA view. (**10C**). Postoperative true lateral view showing retrograde insertion of a single partially threaded IMHCS.

In our series of proximal phalangeal fractures, 39% of patients demonstrated anatomical reduction and good or excellent TAM scores. Analysis of the postoperative true lateral radiographs in this subgroup revealed perfect alignment of the dorsal cortex, with no visible step-offs in all but one patient. A clear lateral view was missing in the latter patient. This configuration ensures an optimal gliding surface for the extensor apparatus, which is essential for achieving full recovery of TAM. Figure 4 displays the lateral views of ten patients from this group, all demonstrating anatomically reduced dorsal cortices.

**Figure 5:**
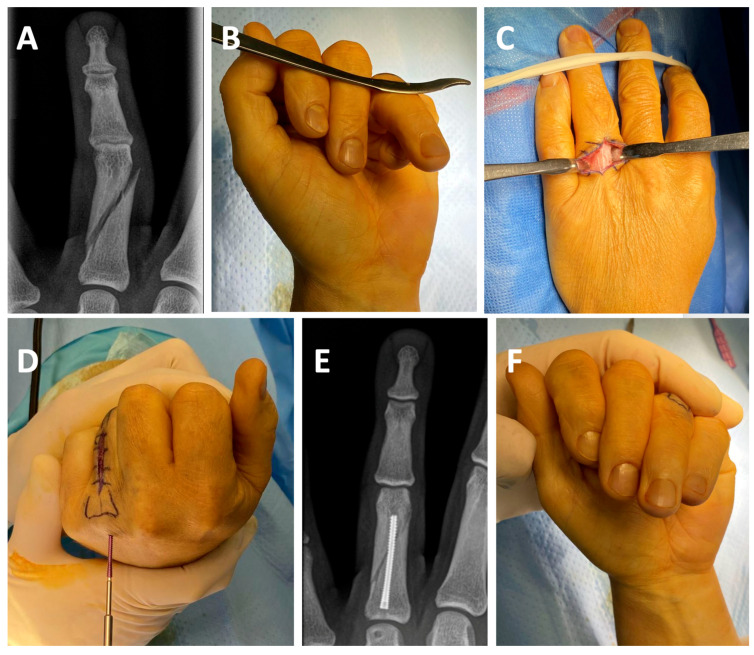
Spiral extra-articular fracture of the proximal phalanx. (**A**). Preoperative anteroposterior radiograph showing a displaced fracture. (**B**). Intraoperative clinical image that demonstrates marked clinodactyly due to malalignment. (**C**). Mini-open dorsal approach through a longitudinal skin incision and midline split of the extensor tendon, enabling direct visualization and accurate fracture reduction. (**D**). Transarticular insertion of a fully threaded intramedullary headless compression screw. (**E**). Postoperative radiograph confirming anatomic fracture reduction and proper positioning of a stabilizing K-wire. (**F**). Intraoperative clinical image after the procedure, demonstrating corrected alignment.

Given these findings, the primary goal in treating proximal phalangeal fractures with IMHCSs should be the restoration of a smooth and anatomically aligned dorsal cortex, as this is essential for preserving tendon gliding and optimal digital function. Anatomical reduction facilitates this outcome and should be pursued where possible. A minimal open approach—as described by Caekebeke et al. (Figure 5)—can be used to achieve accurate dorsal cortical alignment [38].

A limitation of the current study is that it is underpowered to definitively determine whether an association exists between fracture reduction status and functional outcome in metacarpal fractures. A follow-up prospective study may be warranted.

## Figures and Tables

**Table 1:** Metacarpal and proximal phalangeal fracture summary.

Parameter	Metacarpal Group	Proximal Phalanx Group
Number of fractures	41	28
Mean age (years)	35 (range 16–74)	33 (range 12–73)
Sex (male/female)	32/9	18/10
Mean consolidation time (days)	46 (range 23–140)	41 (range 24–89)
Second ray	12% (*n* = 5)	18% (*n* = 5)
Third ray	17% (*n* = 7)	7% (*n* = 2)
Fourth ray	37% (*n* = 15)	18% (*n* = 5)
Fifth ray	34% (*n* = 14)	57% (*n* = 16)
Retrograde insertion	63% (*n* = 26)	14% (*n* = 4)
Antegrade insertion	37% (*n* = 15)	82% (*n* = 23)
Screws per fracture	1 (100%)	1 (71%)–2 (29%)

**Table 2:** Total Active Motion (TAM) method, as adopted by the American Society for Surgery of the Hand (ASSH).

TAM	Flexion Quality Score	Proximal Phalanx TAM (N = 28 Fingers in 24 Patients)	Metacarpal TAM (N = 41 Fingers in 33 Patients)
Excellent (260–270) & Good (250–259)	2	17 (61%)	37 (90%)
Fair (200–249)	1	5 (18%)	3 (7%)
Poor (<200)	0	6 (21%)	1 (2%)

**Table 3:** This contingency table shows the distribution of the TAM scores by fracture reduction status in metacarpal fractures. A reduction score of 0 indicates non-anatomical reduction, and 1 indicates anatomical reduction. No significant association was found (*p* = 0.1303; two-tailed, exact test, effect size Cohen’s w = 0.278).

Reduction	ROM = 0	ROM = 1	ROM = 2
0	2% (*n* = 1)	5% (*n* = 2)	29% (*n* = 12)
1	0% (*n* = 0)	2% (*n* = 1)	61% (*n* = 25)

**Table 4:** This contingency table shows the distribution of TAM scores by fracture reduction status in proximal phalangeal fractures. A reduction score of 0 indicates non-anatomical reduction, and 1 indicates anatomical reduction. A statistically significant association was observed between reduction status and flexion outcome (*p* = 0.0014; two-tailed, exact test, effect size Cohen’s w = 0.802).

Reduction	ROM = 0	ROM = 1	ROM = 2
0	39% (*n* = 11)	11% (*n* = 3)	7% (*n* = 2)
1	0% (*n* = 0)	3% (*n* = 1)	39% (*n* = 11)

## Data Availability

The raw data supporting the conclusions of this article will be made available by the authors upon reasonable request.

## Abbreviations

The following abbreviations are used in this manuscript:

IMHCS	Intramedullary headless compression screw
TAM	Total Active Motion
K-wires PA	Kirschner wires Posteroanterior
